# Laparoscopic posterior Colpotomy for a Cervico-vaginal Leiomyoma: hymen conservative technique.

**Published:** 2016-09

**Authors:** GS Wehbe, M Doughane, R Bitar, Z Sleiman

**Affiliations:** Department of Obstetrics and Gynecology, Lebanese university, Beirut, Lebanon.; Department of Obstetrics and Gynecology, Lebanese American University, Beirut, Lebanon.

**Keywords:** Laparoscopy, colpotomy, hymen, hymenal integrity, leiomyoma

## Abstract

**Background:**

Leiomyomas are the most common benign tumours of the uterus. The diagnosis, evaluation and treatment of prolapsed pedunculated submucous myoma may need vaginal access compromising sometimes the hymenal integrity. Laparoscopic management of a pedunculated submucous myoma in a Middle Eastern virgin patient is described as a safe alternative.

**Case:**

A 20-year-old, nulliparous virgin woman presented to the outpatient clinic for irregular menstrual bleeding of 2 months duration. Imaging revealed a 5×5 cm solid mass in the cervico-vaginal location filling the vagina suggestive of a prolapsed pedunculated submucous leiomyoma. Due to the patient’s desire of preserving her intact hymen, a laparoscopic posterior colpotomy was performed and the mass was removed successfully.

**Conclusion:**

Laparoscopic posterior colpotomy, preserving the hymenal integrity in a virgin patient, provides excellent access and visualization and it is a safe tool for the management of a cervico-vaginal pedunculated submucous myoma by a skilled laparoscopic gynaecologic surgeon.

## Introduction

Leiomyomata are the most common tumours of the uterus and the female pelvis. Before the 20th century, uterine leiomyomata often grew to enormous size and caused massive and sometimes fatal bleeding, pain and emaciation. The development of gynaecologic surgery and anaesthetic skills allowed the safe removal of these tumours. Fibroids may develop anywhere within the muscular wall of the uterus, including submucosal, intramural, or subserosal positions (Rock and Jones, 2008). Removal of pedunculated submucosal fibroid within the uterine cavity is typically performed using hysteroscopy. Infrequently, uterine contractions will push a pedunculated submucosal fibroid through the cervical canal and it may prolapse into the vagina. Vaginal myomectomy is the treatment of choice for prolapsed pedunculated submucous myoma (Golan et al., 2005).

Intact hymen is considered as a sign of sexual purity in some societies especially in the Middle East (Kaivanara, 2016). Therefore, anatomic integrity of the hymen is a major social concern making vaginal access for surgical procedures in virgin patients with intact hymenal ring unacceptable. In this case report, we present the management of a prolapsed pedunculated submucous leiomyoma in a virgin patient

## Case report

A 20-year-old, nulliparous virgin Lebanese girl presented to our private gynaecology clinic for meno-metrorragia of 2 months duration. Associated symptoms included watery yellow-pinkish vaginal discharge, constant menstrual-type cramping, pelvic pressure pain and chronic fatigue. Her previous medical history was uneventful. She has never been sexually active.

On gynaecologic examination, inspection showed normal vulvar area with annular intact hymen. Abdominal examination was unremarkable. Rectal examination revealed significant vaginal fullness. Laboratory tests showed anaemia, as her haemoglobin was 8 g/dl. Pelvic ultrasound revealed a 5×5 cm solid hypo-echoic mass in the cervico- vaginal zone, well delineated, filling the vaginal margins, clearly out of endometrial cavity. Uterus and adnexa were normal. Pelvic MRI confirmed the ultrasound findings and suggested that this mass was most likely to be a prolapsed pedunculated submucous leiomyoma.

Surgical removal of the myoma via vaginal approach was offered to the patient as a first line surgical option. The patient was informed that her hymen integrity can be lost during the procedure and a hymenal repair can be done directly after the termination of the intervention. The patient and her family refused the vaginal approach. Due to the patient’s desire for preserving hymenal integrity, a laparoscopic abdominal approach was performed.

**Fig. 1:**
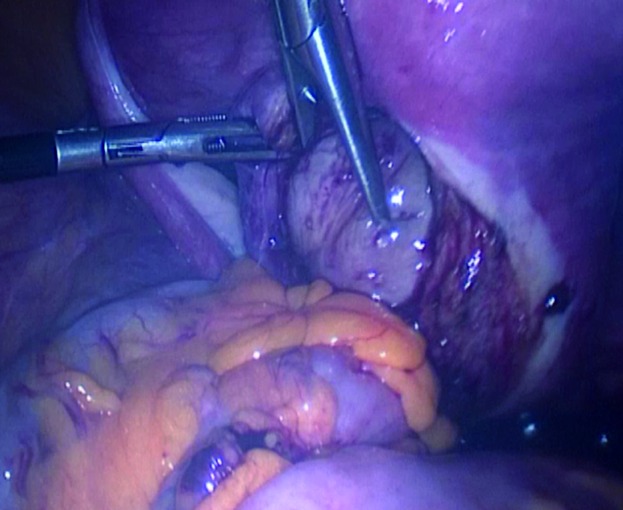
— Posterior colpotomy and identification of the myoma.

**Fig. 2:**
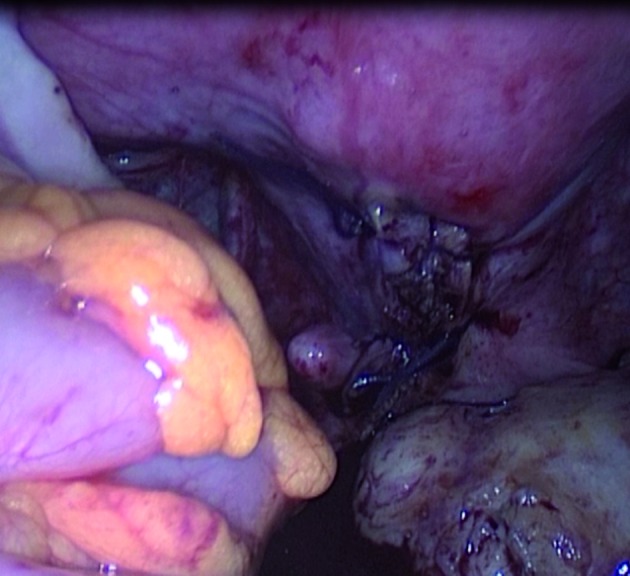
— Posterior colporrhaphy and extracted myoma.

Inspection showed a normal uterus, adnexa and pelvic surfaces. A bulky appearance located under the posterior vaginal wall was noticed. The uterus was sutured to the anterior pelvic wall in order to have better exposure and enough access to the Douglas pouch because the introduction of uterine manipulator was not possible in this virgin patient. A vertical posterior colpotomy was performed. A firm regular pedunculated mass (Figure 1) was identified and delivered through the incision after coagulation using bipolar electrocautery and section of its peduncle. A 5×5 cm mass was removed and extracted by an electrical morcellator. Vaginal defect was repaired with absorbable vicril 0 laparoscopic simple sutures (Figure 2). Histopathologic evaluation showed a benign leiomyoma with degeneration changes. The postoperative course was smooth and she was discharged without any complications 48 hours after the surgery.

## Discussion

Submucous myomas have been implicated in women with abnormal uterine bleeding, infertility, and adverse pregnancy outcomes including multiple pregnancy losses. Classification of submucous leiomyomas should be done when considering surgery as a therapeutic option. Submucous leiomyomas are categorized into three subtypes according to the proportion of the lesion’s diameter that is within the myometrium. Based on the European Society of Gynecological Endoscopy (ESGE) classification system, pedunculated submucous myoma is classified as type 0 submucous fibroid (American Association of Gynecologic Laparoscopists, 2012).

Vaginal myomectomy is the treatment of choice for prolapsed pedunculated submucous myoma with a minimal associated morbidity (Golan et al., 2005).

Virginity, defined as an intact hymen has an undeniable value in the Middle Eastern culture. Failure to prove virginity on the wedding night may have serious consequences for a girl (Kaivanara, 2016).

For many young women, loss of virginity may result with social burden and removing such large masses outside the vagina may pose potential risk for losing virginity. Bleeding during the first intercourse is still a very considerable social value. There are several hymen types, and some of them do not bleed during first intercourse. Thus, sometimes women have to prove their virginity with vaginal examination. In some Arabic countries, the patient has to go to the police to get a written permission before any surgery that may harm the hymen can be performed. A second visit should be done after the surgery to prove that the hymen integrity is intact or restored. It is a limiting factor for gynaecologists in diagnosing and treating such a case. The patient’s desire and informed consent should always be taken into consideration.

Hysteroscopy with the vaginoscopic approach is a feasible quick, and very well tolerated intervention, without analgesia or local anaesthesia (Misra et al., 2005). It is an easy way to gain access to the cervix and the cervical canal and to diagnose and treat vaginal lesions (Di Spiezio Sardo et al., 2016). Few reports discussed the protection of hymenal integrity during operative vagino-hysteroscopy (Xu et al., 2006; Küçük, 2007; Ou et al., 2009). The hysteroscopic approach is usually successful for resection of small pedunculated submucous myomas. However, this technique is limited while operating on large masses. In our case, the size of the myoma was thought to harm hymenal integrity while removing mini-laparotomic anterior colpotomy has been recently described as a hymenal integrity sparing technique for the excision of a cervico-vaginal pedunculated submucous myoma in a virgin lady (Yalçin et al., 2016). In the presence of a skilled laparoscopic surgeon, a minimal invasive approach is a safe alternative to laparotomy with better outcomes.

The management of benign and malignant pelvic gynaecological diseases using laparoscopy is feasible and safe (Vásquez-Ciriaco et al., 2016). Minimally invasive surgery has a significant number of advantages including a reduction in the post-operative pain and cosmetic injury, early ambulation, shorter hospital stay and a rapid return to normal activities (Coventry, 1995). Due to social and cultural considerations we can extend the indications of laparoscopy to profit from its benefits and respect the social context. Laparoscopic myomectomy for a cervico-vavinal prolapsed pedunculated large submucous myoma in a virgin lady can be a new indication.

In conclusion, hysteroscopy remains the “gold standard” intervention in the management of symptomatic submucous pedunculated fibroids. However, different factors such as myoma dimensions, anatomic obstacles or patient’s consideration regarding her hymenal integrity limit this approach. In our traditional oriental culture, the prevention of hymen ring injury during surgery should be highly considered before proceeding with any kind of intervention. As an alternative, posterior colpotomy and myomectomy via laparoscopic access seems feasible when hymenal integrity is important. However this approach requires adequate equipment and advanced laparoscopic surgical skills.
